# The Chemokine Receptor CCR5 Links Memory CD4^+^ T Cell Metabolism to T Cell Antigen Receptor Nanoclustering

**DOI:** 10.3389/fimmu.2021.722320

**Published:** 2021-12-07

**Authors:** Raquel Blanco, Marta Gómez de Cedrón, Laura Gámez-Reche, Ana Martín-Leal, Alicia González-Martín, Rosa A. Lacalle, Ana Ramírez de Molina, Santos Mañes

**Affiliations:** ^1^ Department of Immunology and Oncology, Centro Nacional de Biotecnología (CNB/CSIC), Madrid, Spain; ^2^ Precision Nutrition and Cancer Program, Molecular Oncology Group, IMDEA Food Institute, CEI UAM+CSIC, Madrid, Spain; ^3^ Department of Biochemistry, Universidad Autónoma de Madrid, and Instituto de Investigaciones Biomédicas Alberto Sols (IIB/CSIC), Madrid, Spain

**Keywords:** memory CD4+ T cells, efector CD4+ T cells, metabolic reprogramming, chemokine signaling, CCR5, BCL6, glycolysis

## Abstract

The inhibition of anabolic pathways, such as aerobic glycolysis, is a metabolic cornerstone of memory T cell differentiation and function. However, the signals that hamper these anabolic pathways are not completely known. Recent evidence pinpoints the chemokine receptor CCR5 as an important player in CD4^+^ T cell memory responses by regulating T cell antigen receptor (TCR) nanoclustering in an antigen-independent manner. This paper reports that CCR5 specifically restrains aerobic glycolysis in memory-like CD4^+^ T cells, but not in effector CD4^+^ T cells. CCR5-deficient memory CD4^+^ T cells thus show an abnormally high glycolytic/oxidative metabolism ratio. No CCR5-dependent change in glucose uptake nor in the expression of the main glucose transporters was detected in any of the examined cell types, although CCR5-deficient memory cells did show increased expression of the hexokinase 2 and pyruvate kinase M2 isoforms, plus the concomitant downregulation of Bcl-6, a transcriptional repressor of these key glycolytic enzymes. Further, the TCR nanoclustering defects observed in CCR5-deficient antigen-experienced CD4^+^ T cells were partially reversed by incubation with 2-deoxyglucose (2-DG), suggesting a link between inhibition of the glycolytic pathway and TCR nanoscopic organization. Indeed, the treatment of CCR5-deficient lymphoblasts with 2-DG enhanced IL-2 production after antigen re-stimulation. These results identify CCR5 as an important regulator of the metabolic fitness of memory CD4^+^ T cells, and reveal an unexpected link between T cell metabolism and TCR organization with potential influence on the response of memory T cells upon antigen re-encounter.

## Introduction

The adaptive immune system has the ability to generate immunological memory. This allows for rapid and robust secondary responses upon antigen re-encounter (1). The response of memory T (T_M_) cells to low antigen concentrations has been linked to the antigen-independent formation of T cell antigen receptor (TCR) oligomers known as nanoclusters (2–4). The nanoscopic organization of the TCR molecules is not exclusive to T_M_ cells; it also occurs in effector T (T_E_) cells, although to a lesser extent. Indeed, the antigenic sensitivity gradient in CD4^+^ and CD8^+^ T cell subsets (T_M_>T_E_>>naive) correlates with the valency of TCR nanoclusters at the cell surface (5, 6). TCR nanoscopic organization allows cooperativity between TCR molecules (7) and increases avidity for multimeric peptide-major histocompatibility complexes (5, 8). TCR nanoclustering in antigen-experienced T_M_ and T_E_ lymphoblasts is strongly dependent on the sterol and sphingolipid composition of the plasma membrane (6, 9, 10); the importance of the cell’s metabolic state in TCR organization has, however, been left completely unexplored.

A solid body of evidence indicates that dynamic changes in cellular metabolism determine the functionality and fate of CD4^+^ and CD8^+^ T cells (11–13). Each differentiation state and lineage subset of T cells has a unique metabolic profile. Quiescent, naive T cells, which have low metabolic needs, are largely dependent on the oxidative phosphorylation (OXPHOS) of small amounts of glucose or fatty acids in the mitochondria to generate ATP (12). Upon antigen stimulation, T_E_ cells increase both OXPHOS and aerobic glycolysis to fulfill their new ATP and building block demands. The net result is a metabolic switchover that reduces the OXPHOS/glycolytic ratio in these cells. This increase in glycolysis, which occurs in CD8^+^ and all effector CD4^+^ T helper (Th) subsets (Th1, Th2 and Th17), is not only important for generating the biomass needed for the expansion of activated cells, it is also required for the expression of cytokines involved in T cell effector function (14–17). In contrast, the metabolism of CD4^+^ regulatory T cells, and of both CD4^+^ and CD8^+^ T_M_ cells, largely relies on OXPHOS and fatty acid oxidation (FAO) (12, 16, 18). Thus, T_M_ cells undergo a metabolic switchover that increases the OXPHOS/glycolytic ratio compared to T_E_ cells. Indeed, the inhibition of glycolysis is a necessary step in preserving the generation of long-lived CD8^+^ T_M_ cells, whereas enhanced glycolytic flow prevents T_M_ cell formation (19).

Switches between these dynamic metabolic programs are orchestrated by the TCR plus co-stimulatory and cytokine signaling. Together, these induce top-down signaling circuits culminating in the expression or repression of specific transcription factors. For instance, interleukin (IL)-2 signaling boosts the transcription of glycolytic genes through the induction of interferon-regulatory factor (IRF)-4, hypoxia-inducible factor (HIF)-1α, and Myc (20). Interestingly, IL-2 also downregulates the glycolysis-inhibiting repressor B-cell lymphoma 6 (Bcl)-6, thus preventing the induction of a Bcl-6-guided transcriptional program more compatible with T_M_ cell metabolism (21). Other cytokines such as IL-15 and IL-7 promote T_M_ cell formation by triggering mitochondrial oxidative metabolism through the expression of carnitine palmitoyltransferase-1a (CPT1a) and lipases, which respectively control FAO and the mobilization of intrinsic fatty acids (22). Co-stimulatory and cytokine signaling also control mitochondrial fusion and fission dynamics, and the ultrastructure of the cristae, affecting mitochondrial respiratory capacity and hence the formation of T_E_ and T_M_ cells (23–27). Although information on the regulation of T cell metabolism has increased in recent years, much remains to be learnt about the array of signals involved in its reprogramming.

T cells are also exposed to signals provided by chemokines - cytokines that act through seven-transmembrane G-protein-coupled receptors and traditionally catalogued as regulators of leukocyte trafficking (28). Solid evidence exists, however, that some chemokine receptors influence T-cell fate and function in a chemotactic-independent manner (29). In particular, C-C chemokine receptor 5 (CCR5) has been implicated in maximizing CD4^+^ T cell co-stimulation and the induction of transcriptional programs responsible for cytokine production (30–34). CCR5 transduces signals from CCL3, CCL4 and CCL5, the expression of which is induced upon TCR-mediated activation (35). Recently, CCR5 has been involved in CD4^+^ T_M_ cell responses. CCR5 deficiency does not affect CD4^+^ T_M_ cell generation, but reduces TCR nanoclustering organization and, consequently, the antigenic sensitivity of T_M_ cells after antigen re-encounter (6). As a result, CCR5^-/-^ mice show impaired production of high-affinity class-switched antibodies after antigen re-challenge, a phenomenon dependent on CD4^+^ T_M_ cell function.

Since CCR5 activity affects CD4^+^ T_M_ cell functionality, the present work examines whether it also affects T_M_ cell metabolism. The information of potential effects of CCR5 on T cell metabolism is, however, limited. In activated CD4^+^ T_E_ cells, CCR5 has been reported to increase glucose uptake, glycolysis and AMP-activated protein kinase (AMPK)-α1 activity and, therefore, presumably FAO; indeed, the inhibition of glycolysis and the AMPK pathway prevents CCR5-mediated chemotaxis (36). The CCL5-mediated chemotactic activity of activated and resting CD4^+^ T_M_ cells has different metabolic requirements (37), but the involvement of CCR5 activation in metabolic alterations has never before been studied. The present work shows that CCR5 deficiency enhances glycolysis in CD4^+^ T_M_ cells, thus hindering the metabolic switch associated with the T_M_ cell lineage. OXPHOS and glycolysis were found comparable in CCR5-deficient and CCR5-proficient CD4^+^ T_E_ cells, indicating the CCR5 effect in T_M_ cells to be specific to them. CCR5-deficient CD4^+^ T_M_ cells showed very little expression of Bcl-6, whereas some key enzymes regulating the glycolytic flow were increased. Strikingly, the inhibition of glycolysis in CCR5-deficient, antigen-experienced CD4^+^ T lymphoblasts increased the degree of TCR nanoclustering. These results indicate that CCR5 improves the functional and metabolic fitness of CD4^+^ T_M_ cells, and reveal an unexpected bond between metabolic reprogramming and TCR nanoscopic organization.

## Materials and Methods

### Mice

TCR transgenic OT-II CCR5^-/-^ mice (31) recognizing the peptide OVA_323–339_ (ISQAVHAAHAEINEAGR; I-Ab MHC class II molecule) were maintained under specific-pathogen-free conditions at the CNB animal facilities, in agreement with Spanish national and EU guidelines. All animal procedures were approved by the ethics committees of the CNB and the *Comunidad de Madrid* (PROEX 277/14; PROEX 090/19).

### Culture of Mouse Primary T Cells

Spleen and lymph nodes from 6 to 12 week-old OT-II WT and CCR5^-/-^ mice were isolated and cell suspensions obtained using 40 µm pore filters. Erythrocytes were lysed with AKT lysis buffer (0.15 M NH_4_Cl, 10 mM KHCO_3_, 0.1 mM EDTA) and activated with the OVA_323–339_ peptide for 3 days in complete medium, i.e., RPMI 1640 (Biowest), 10% FBS, 1 mM sodium pyruvate, 2 mM L-glutamine, 1% non-essential amino acids, 100 U/ml penicillin/streptomycin, 10 µM β-mercaptoethanol. The antigen was removed and the cells cultured with IL-2 (5 ng/ml) or IL-15 (20 ng/ml) for four more days.

### Analysis of Cell Bioenergetics

The oxygen consumption rate (OCR) and the extracellular acidification rate (ECAR) were measured using an XF96 Extracellular Flux analyzer (Seahorse Bioscience). CD4^+^ T_E_/T_M_ cells were seeded (0.3x10^6^ cells/well) on a XF96 cell culture microplate previously treated with Cell-Tak (Corning). Mitochondrial stress tests were performed by incubating cells for 1 h in the absence of CO_2_ in non-buffered XF assay medium pH7.4 (Seahorse Bioscience), supplemented with 25 mM glucose, 2 mM glutamine and 1 mM sodium pyruvate. After basal rate measurements, different modulators of mitochondrial respiration were injected sequentially (1): 2.5 μM oligomycin to inhibit ATP-synthase and to calculate the ATP-linked oxygen consumption (2); 1.5 μM carbonyl cyanide-P-trifluoro-methoxy-phenylhydrazone (FCCP; an uncoupling agent) to obtain the maximum respiration under stress conditions; and (3) a mix of 0.5 μM rotenone/antimycin A to completely block mitochondrial respiration by inhibiting complexes I and III respectively.

The oxidation of exogenous fatty acids was measured using palmitate-BSA as a substrate. Briefly, cells were seeded overnight in complete medium. Forty-five minutes prior to the assay, cells were incubated with non-buffered XF assay medium pH7.4 (Seahorse Bioscience) supplemented with palmitate-BSA, 1 mM glucose, 0.5 mM carnitine, and 5 mM HEPES, adjusted to pH 7.4, and incubated (30-45 min, 37°C) in a non-CO_2_ incubator; etomoxir (40 μM) was added in the corresponding wells 15 min before starting the assay. OCR was measured under basal conditions and after the sequential addition of 2.5 μM oligomycin, 1.5 μM FCCP and 0.5 μM rotenone/antimycin A.

Glycolysis stress tests were performed using cells starved in a non-CO_2_ incubator for 1 h at 37°C in non-buffered XF assay medium (Seahorse Bioscience) supplemented with 2 mM glutamine and 1 mM sodium pyruvate. After measuring basal ECAR and OCR, 15 mM glucose were injected to stimulate glycolysis, followed by 2.5 μM oligomycin to obtain the maximum glycolytic capacity *via* the inhibition of oxygen consumption. Finally, 100 mM of 2-deoxy-D-glucose (2-DG) were injected to shut down glycolysis. For glycolytic rate assays, cells were incubated in the absence of CO_2_ for 1 h in non-buffered XF assay medium (Seahorse Bioscience) supplemented with 25 mM glucose, 2 mM glutamine and 1 mM sodium pyruvate. After basal ECAR measurements, 0.5 μM rotenone/antimycin and 100 mM 2-DG were injected.

OCR and ECAR were measured three times after the addition of each drug. At least three animals per condition, run in triplicate, were used in each experiment. Calculations were performed with the Seahorse XF Cell Test Report Generator software (Seahorse Bioscience).

### Chemokine Determination

The supernatant of OT-II cells (10^6^ cells/well) differentiated under T_E_ and T_M_ conditions (as indicated above) was collected on day 7 of culture, and mouse CCL3, CCL4 and CCL5 levels determined using a specific sandwich ELISA (R&D Systems) following the manufacturer’s instructions.

### Quantitative Real-Time PCR

Total RNA was extracted from cells using the RNeasy Mini Kit (Qiagen), and cDNA synthesized from 1 μg total RNA using the High Capacity cDNA Reverse Transcription Kit (Promega). Quantitative RT–PCR was performed in a QuantStudio 5 Real-Time PCR System (Applied Biosystems), using FluoCycle II SYBR Master Mix (EuroClone) with the primer pairs listed in **Supplementary Table S1**. Gene expression was normalized using the 18S ribosomal RNA signal.

### Western Blot Analysis

Protein extracts were obtained after cell lysis with RIPA buffer (50 mM Tris-HCl pH 8.0, 150 mM NaCl, 1% NP-40, 0.5% sodium deoxycholate and 1% SDS) supplemented with protease and phosphatase inhibitors (1 mM PMSF, 1 mM Na_3_VO_4_, 10 μg/ml leupeptin, 10 μg/ml aprotinin and 5 mM NaF). Proteins were quantified using the Micro BCA Protein Assay Kit (Pierce). Equal amounts of proteins were resolved on 10% polyacrylamide gels and transferred to PVDF membranes. Membranes were probed with anti-Bcl-6 (BD Pharmingen) and anti-β-actin (Sigma Aldrich) antibodies.

### Flow Cytometry Analysis

WT and CCR5^-/-^ OT-II lymphoblasts (10^6^) differentiated under T_E_ or T_M_ conditions were stained (30 min, 4°C) with LIVE/DEAD™ Fixable Near-IR Dead Cell Stain (Thermofisher Scientific) and, after extensive washing with ice-cold PBS-2% BSA, stained (15 min, 4°C) with anti-CD4-PECy5.5 (clone GK1.5; BioLegend) in the dark. The cells were then fixed and permeabilized using the eBioscience™ Foxp3/Transcription Factor Fixation/Permeabilization Kit (Thermofisher Scientific) following the manufacturer’s instructions, and stained (1 h, 20°C, in the dark) with anti-Bcl6-PE (clone K112-91; 2 µg/ml; BD Bioscience). Cells were analyzed using a Gallios Flow Cytometry Analyzer (Beckman Coulter), and data processed using FlowJo software (BD Bioscience).

### Glucose Internalization

Glucose uptake was determined for the whole cell population using the Glucose Uptake-Glo Assay (Promega). Basically, WT and CCR5^-/-^ OT-II lymphoblasts differentiated under T_E_ or T_M_ conditions (0.5x10^6^) were starved for 30 min in glucose-free RPMI 1640 medium (Biowest) supplemented with 2 mM L-glutamine, 1% non-essential amino acids and 100 U/ml penicillin/streptomycin, and then incubated with 2-DG (20 min, 37°C). Cells were lysed and 2-DG incorporation determined following the manufacturer’s instructions.

### Immunogold Labeling, Replica Preparation, and EM Analysis

CCR5^-/-^ and WT OT-II lymphoblasts treated - or not - with 2-DG (2 mM, 24 h) were used to prepare cell surface replicas as previously described (5, 6). Briefly, T cells were fixed in 1% paraformaldehyde and labeled with anti-mouse CD3ϵ mAb (145-2C11), followed by 10 nm gold-conjugated protein A (Sigma-Aldrich). Labeled cells were adhered to poly-L-lysine-coated mica strips and fixed with 0.1% glutaraldehyde. Samples were covered with another mica strip, frozen in liquid ethane (KF-80, Leica), and stored in liquid nitrogen. Cell replicas were prepared with a Balzers 400T freeze fracture (FF) unit, mounted on copper grids, and analyzed using a JEM1010 electron microscope (Jeol) operating at 80 kV. Images were taken with a Bioscan CCD camera (Gatan) and processed with TVIPS software. EM image acquisition and quantification was performed by two researchers, one of them blind to the experiment. The number of TCR molecules in the same cluster was determined when the distance between gold particles was smaller than their diameter (10 nm).

### Re-Stimulation Assays

CCR5^-/-^ OT-II lymphoblasts were treated with vehicle or 2-DG (2mM) for 24 h and, after extensive washing to remove all remnants of 2-DG, co-cultured (24 h) with irradiated (15 Gy) splenocytes loaded (2 h, 37°C) with different concentration of OVA_323-339_ peptide. Supernatants were collected to measure IL-2 by ELISA (ELISA MAX Deluxe, BioLegend). Proliferation was assessed *via* methyl-^3^[H]-thymidine (1 μCi/well) incorporation into DNA, using a 1450 Microbeta liquid scintillation counter (PerkinElmer).

### Statistical Analysis

Statistical analyses were performed using Prism software (GraphPad). Differences were assessed using the two-tailed Student *t* test with Welch’s correction or the Holm-Sidak correction for multiple *t* test comparisons, or, when appropriate, two-way ANOVA with the *post-hoc* Bonferroni test for multiple comparisons. The Chi-square test was used to analyze the overall distribution of gold particles. Variances were compared using the F test. Data are expressed as means ± SEM. Significance was set at p<0.05.

## Results

### CCR5 Does Not Alter Mitochondrial Activity in CD4^+^ T Cells

**Figure 1:**
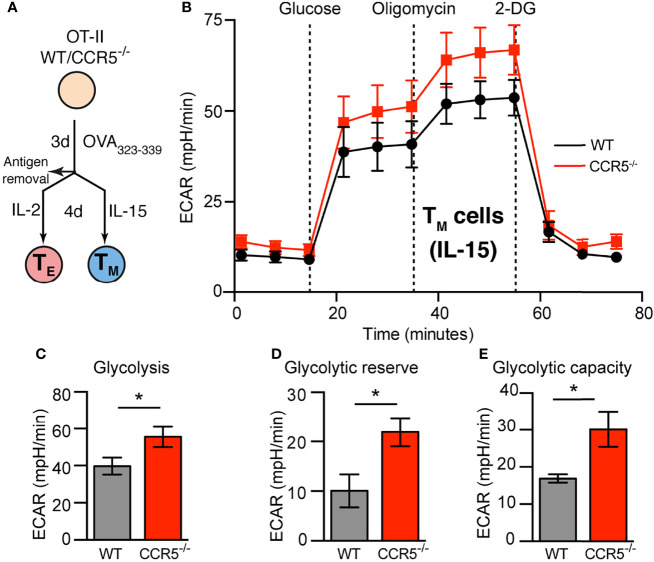
CCR5 deficiency increases glycolytic metabolism in memory CD4^+^ T cells. **(A).** Diagram showing the *ex-vivo* activation and differentiation of primary CD4^+^ T cells (OT-II). **(B)** ECAR profiles of WT and CCR5^-/-^ T_M_ (IL-15-expanded) lymphoblasts differentiated as in **(A)**, incubated in XF assay medium supplemented with 2 mM glutamine and 1 mM sodium pyruvate in the absence of CO_2_, and subsequently inoculated with glucose, oligomycin and 2-DG as indicated. **(C–E).** Determination of glycolysis **(C)**, glycolytic reserve **(D)** and glycolytic capacity **(E)** (details in *Results*) from the ECAR curves obtained as in **(B)**. Data represent means ± SEM (n≥9 from three independent experiments). *p < 0.05, two-tailed Student *t* test.

To analyze whether CCR5 affects the metabolic reprogramming of CD4^+^ T cells, WT and CCR5^-/-^ OT-II splenocytes were stimulated with cognate antigen and subsequently cultured for four days in the presence of IL-2 or IL-15, but in the absence of antigenic stimulation (**Figure 1A**). In accordance with other studies (6, 23), these culture conditions generated resting CD4^+^ T_E_ (IL-2) or T_M_ (IL-15) lymphoblasts, respectively. Notably, T_E_ and T_M_ lymphoblasts expressed comparable levels of the main CCR5 ligands (CCL3, CCL4 and CCL5) and the receptor CCR5 (**Supplementary Figure S1**), suggesting that the differentiation conditions do not affect potential autocrine/paracrine CCR5 signaling (6).

The oxygen consumption rate (OCR) was monitored as an indicator of mitochondrial function in WT and CCR5^-/-^ T_E_ and T_M_ cells, using glucose as carbon source. No differences were seen between any cell type in terms of basal or maximum OCR (OCRmax), spare respiratory capacity (SRC; a variable determining the capacity of the cell to respond to an energy demand), or ATP production (**Supplementary Figure S2**). To rule out that the lack of differences was associated with the carbon source, similar experiments were performed using palmitate in a low glucose medium (1 mM). Pre-treatment of the cells with etomoxir, a CPT1a inhibitor, drastically reduced the OCR (**Supplementary Figure S3**), allowing the contribution of FAO to the measured variables to be distinguished. Again, no significant differences were seen between any cell type in terms of OCRmax, SRC or ATP production (**Supplementary Figure S3**). In agreement with the lack of differences in OXPHOS determinations between CCR5 proficient and deficient cells, no relative differences were found in the expression of mitochondrial genes (**Supplementary Figure S4**), including NADH:ubiquinone oxidoreductase subunit A9 (NDUFA9, respiratory complex I), succinate dehydrogenase complex iron sulfur subunit B (SDHB; complex II), cytochrome C Oxidase assembly factor heme A:farnesyltransferase (COX10, complex IV), mitochondrial ATP synthetase (ATP5A1; complex V), mitochondrial pyruvate carrier (MPC)-1, or carnitine palmitoyltransferase 1A (CPT-1A). Nevertheless, NDUFA9 and SDHB were upregulated in T_M_ compared to T_E_ cells, independent of CCR5 status. Thus, the absence of CCR5 does not alter mitochondrial activity in CD4^+^ T cells.

### CCR5 Restrains Glycolysis Specifically in CD4^+^ T_M_ Cells

Whether CCR5 affects the glycolytic pathway was next studied, measuring the extracellular acidification rate (ECAR). CCR5 affected none of the glycolytic variables measured in T_E_ cells (**Supplementary Figure S5**), i.e., no significant differences were found between WT and CCR5^-/-^ CD4^+^ T_E_ cells in their glycolytic rate (ECAR after the addition of saturating amounts of glucose), glycolytic capacity (maximum ECAR after oligomycin-induced OXPHOS inhibition) or glycolytic reserve (the capacity to respond to an energetic demand). In contrast, CCR5^-/-^ T_M_ cells showed a significant increase in all the glycolytic variables measured compared to their WT counterparts (**Figures 1B–E**). This suggests that CCR5 signals restrain the glycolytic activity of T_M_ cells.

**Figure 2:**
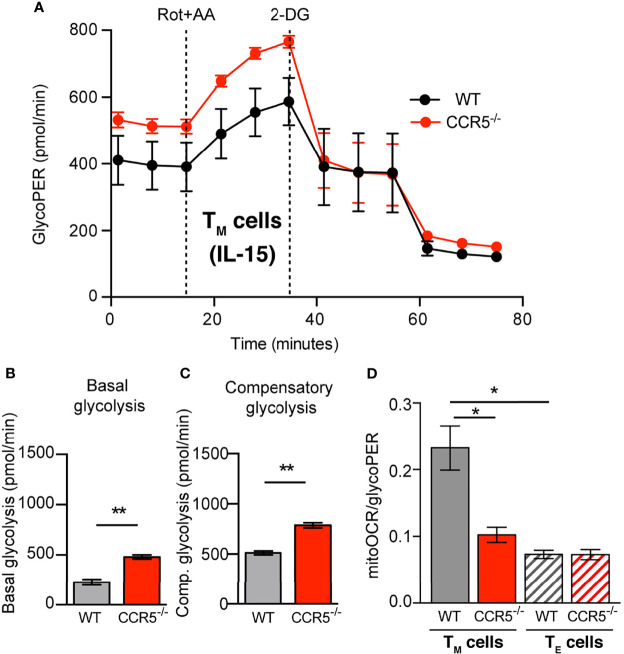
CCR5 deficiency prompts glycolytic metabolism over OXPHOS in memory CD4^+^ T cells. **(A)** GlycoPER profiles in WT and CCR5^-/-^ T_M_ cells under basal condition (non-buffered XF assay medium pH 7.4, containing 25 mM glucose and 2 mM glutamine 1 mM sodium pyruvate, in the absence of CO_2_), and following addition of rotenone/antimycin A (Rot+AA) and 2-DG. **(B, C)** Basal glycolysis **(B)** and compensatory glycolysis **(C)** determined from profiles as in **(A)**. **(D)** Basal mitoOCR/glycoPER ratio in WT and CCR5^-/-^ T_M_ (solid bars) and T_E_ (hatched bars) cells. Data are means ± SEM (n≥9 from three independent experiments). **p < 0.01, *p < 0.05, two-tailed Student *t* test **(B, C)**, or two-way ANOVA with Bonferroni *post-hoc* test **(D)**.

Although extracellular acidification is mainly driven by glycolytic activity, it can also be the consequence of other metabolic processes, such as the production of CO_2_ by the tricarboxylic acid (TCA) cycle. The relative contribution of glycolysis and the TCA cycle activity to ECAR was therefore measured using the glycolytic rate assay. This assay determines the glycolytic proton efflux rate (glycoPER), which represents acidification due solely to glycolysis. Both the WT and CCR5^-/-^ T_E_ cells showed comparable glycoPER values under untreated conditions (basal glycolysis), and after the addition of rotenone/antimycin A to inhibit mitochondrial activity (compensatory glycolysis; **Supplementary Figure S6**). However, the values recorded for both basal and compensatory glycolysis were significantly higher in CCR5^-/-^ than in CCR5-proficient T_M_ cells (**Figures 2A–C**). Further, CCR5 deficiency in T_M_ cells caused a significant reduction in OXPHOS/glycolytic activity (i.e., the mitoOCR/glycoPER ratio) compared to the WT T_M_ cells (**Figure 2D**). Indeed, whereas the WT T_M_ cells showed a higher mitoOCR/glycoPER ratio than did the WT T_E_ cells, the CCR5^-/-^ T_M_ and T_E_ cells showed comparable mitoOCR/glycoPER ratios (**Figure 2D**). Together, these results indicate that the metabolic switch associated with CD4^+^ T_M_ cell formation is impaired in CCR5^-/-^ cells due to their enhanced glycolytic metabolism.

### CCR5 Does Not Affect Glucose Uptake in CD4^+^ T_M_ Cells

**Figure 3:**
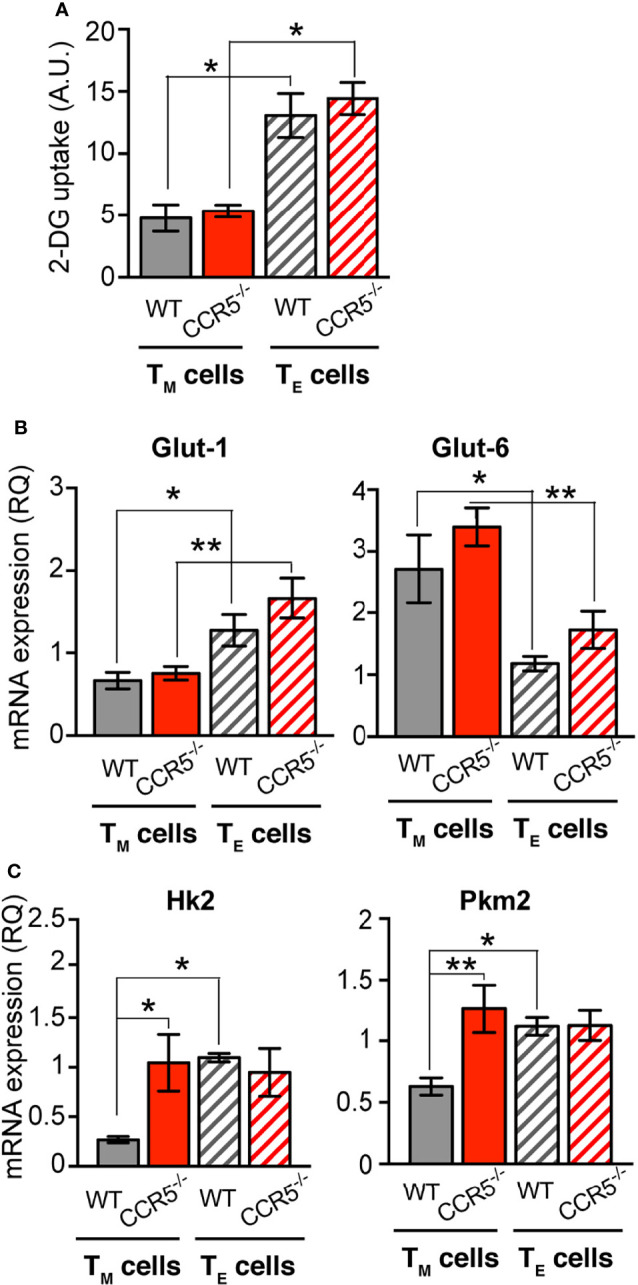
CCR5 regulates glucose uptake and rate-limiting glycolytic enzymes specifically in CD4^+^ T_M_ cells. **(A)** Determination of 2-DG uptake in WT and CCR5^-/-^ T_M_ (solid bars) and T_E_ (hatched bars) cells (*n*=3). **(B, C)** Relative mRNA expression of the glucose transporters Glut-1 and Glut-6 **(B)**, and the glycolytic enzymes Hk2 and Pkm2 **(C)** in WT and CCR5^-/-^ T_M_ and T_E_ lymphoblasts (*n*=12). Data are means ± SEM. **p < 0.01, *p < 0.05, two-way ANOVA with Bonferroni *post-hoc* test.

Given that the CCR5^-/-^ T_M_ cells were substantially more glycolytic than CCR5-proficient cells, a detailed inspection was made of the different elements involved in the glycolytic route. Glucose internalization in WT and CCR5^-/-^ T_E_ and T_M_ cells was examined using 2-DG as a probe, and an increased uptake of glucose was seen in the T_E_ cells but of both backgrounds. This is consistent with the reduced glycolysis seen in the T_M_ compared to the T_E_ cells. Nonetheless, differences in 2-DG internalization were not associated with CCR5 expression (**Figure 3A**).

The expression of glucose transporters was also determined at the mRNA level. Glut-1, Glut-3, Glut-6 and Glut-8 mRNA were detected in WT and CCR5^-/-^ T_E_ and T_M_ cells. Glut-1 and Glut-6 mRNAs were differentially expressed between T_E_ and T_M_ cells, independent of their WT or CCR5^-/-^ genetic background (**Figure 3B**). The mRNA levels for Glut-3 and Glut-8 were associated with no remarkable differences between the cells of any type (**Supplementary Figure S7A**). Collectively, these results indicate that CCR5 does not affect the expression of glucose transporters, which is consistent with the lack of difference seen in glucose uptake between the WT and CCR5^-/-^ cells.

### CCR5 Downregulates Specific Glycolytic Genes in CD4^+^ T_M_ Cells

To study how CCR5 expression regulates the glycolytic flow in T_M_ cells, mRNA levels were determined in WT and CCR5^-/-^ T_E_ and T_M_ cells for enzymes proposed to be rate-limiting in the glycolytic cascade: hexokinase 2 (HK2) and isoform M2 of pyruvate kinase (PKM2) (38). In addition, the relative expression of glyceraldehyde-3-phosphate dehydrogenase (GAPDH) and lactate dehydrogenase (LDHA) was determined. The mRNA levels for HK2 and PKM2 were significantly upregulated in CCR5^-/-^ CD4^+^ T_M_ cells compared to their WT counterparts, but both enzymes were equally expressed in CCR5^-/-^ and WT T_E_ cells (**Figure 3C**). Moreover, both HK2 and PKM2 mRNA levels were higher in the WT T_E_ than in the WT T_M_ cells (**Figure 3C**), confirming the existence of the T_E_/T_M_ metabolic switchover. The expressions of GAPDH and LDHA were not affected by CCR5 status in either the T_E_ and T_M_ cells (**Supplementary Figure S7B**). These results indicate that CCR5 activity in T_M_ cells affects the regulation of key glycolytic enzymes, explaining the glycolytic differences observed between CCR5-proficient and -deficient T_M_ cells.

### Differential Expression of Bcl-6 in WT and CCR5^-/-^ T_M_ Cells

**Figure 4:**
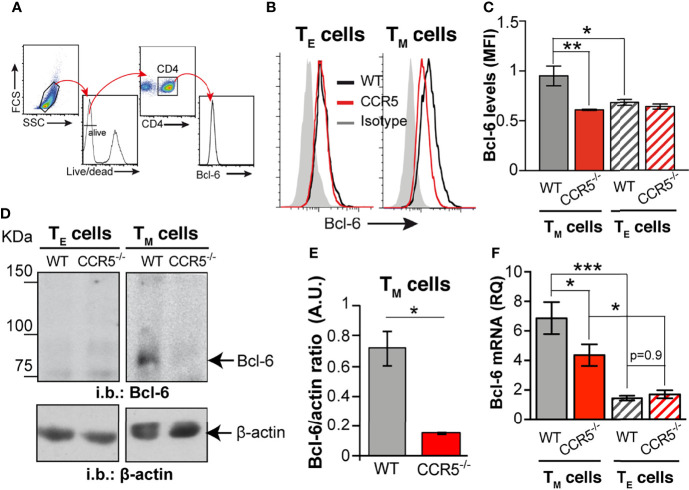
CCR5 deficiency downregulates the glycolytic repressor Bcl-6 in CD4^+^ T_M_ cells. **(A)** Gating strategy for determination of relative Bcl-6 levels by flow cytometry. **(B)** Representative histograms for Bcl-6 levels in WT (black lines) and CCR5^-/-^ (red lines) T_E_ (left) and T_M_ (right) cells; the isotype control histogram is also shown (gray). **(C)** Quantification of the mean fluorescent intensity (MFI) for Bcl-6 staining in WT and CCR5^-/-^ T_E_ (hatched bars) and T_M_ (solid bars) cells (*n*=3) **(D)**. Representative immunoblots of Bcl-6 and β-actin (loading control) in total cell extracts from WT and CCR5^-/-^ T_E_ and T_M_ cells (*n*=3). **(E)** Bcl-6: β-actin densitometry ratio from immunoblots as in **(A)**. **(F)** Relative expression of *Bcl-6* mRNA in WT and CCR5^-/-^ T_E_ (hatched bars) and T_M_ (solid bars) cells (*n*=12). Data are means ± SEM. ***p < 0.001, **p < 0.01 *p < 0.05, two-tailed Student *t* test **(E)**, or two-way ANOVA with Bonferroni *post-hoc* test **(C, F)**.

The mechanism behind the deregulation of glycolysis in CCR5^-/-^ T_M_ cells was next investigated. Since glycolysis is not affected by CCR5 in T_E_ cells, it was reasoned that the glycolytic differences between in CCR5^-/-^ and WT CD4^+^ T_M_ cells might be associated with the different cytokines used in T_E_ and T_M_ cell differentiation. Bcl-6 is a transcriptional repressor of genes involved in glycolysis, the expression of which is increased in CD4^+^ T cells cultured under low IL-2 concentrations (21). In agreement with published data, Bcl-6 protein was very low in IL-2-differentiated T_E_ cells, independent of the CCR5 genotype (**Figures 4A–E**). In contrast, Bcl-6 was clearly upregulated in the WT T_M_ cells but remained low in the CCR5^-/-^ T_M_ cells, both by flow cytometry and immunoblotting (**Figures 4A–E**). In agreement with the protein data, Bcl-6 mRNA levels were also significantly higher in the WT than in the CCR5^-/-^ T_M_ cells (**Figure 4F**), whereas no differences were seen between the WT and CCR5^-/-^ T_E_ cells. These results suggest that CCR5 deficiency downregulates Bcl-6 expression at the transcriptional and translational level.

### Glycolytic Repression Promotes TCR Nanoclustering

**Figure 5:**
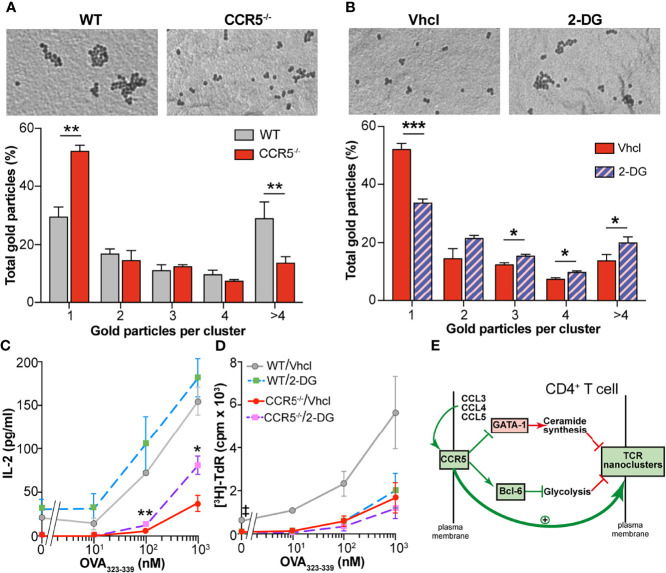
Inhibition of glycolysis increases TCR nanoclustering in CCR5^-/-^ cells. **(A)** Analysis of TCR nanoclustering by EM in OT-II WT and CCR5^-/-^ lymphoblasts (WT, *n*=5 cells, 11,339 particles; CCR5^-/-^, *n*=5 cells, 9,347 particles). Top: A representative small field image shows gold particle distribution in the cell surface replicas of anti-CD3ϵ-labeled cells. Bottom: quantification (means ± SEM) of gold particles in clusters of the indicated size in WT (gray) and CCR5^-/-^ cells (red). **(B)** OT-II CCR5^-/-^ lymphoblasts were expanded in the presence of IL-2 (see Figure 1A), and incubated with the glycolysis inhibitor 2-DG over the last 24 h of expansion (day 7). Lymphoblast surface replicas were stained with anti-CD3ϵ and TCR nanoclustering analyzed by EM. Top: representative small field images show gold particle distribution in the cell surface replicas. Bottom: quantification (means ± SEM) of gold particles in clusters of the indicated size in vehicle- (solid red; *n*=5, 9,347 particles) and 2-DG-treated cells (hatched violet; *n*=5, 11,993 particles). Data are means ± SEM. ***p < 0.001, **p < 0.01, *p < 0.05 one-tailed Student *t* test **(A, B)**. **(C, D)** OT-II WT and CCR5^-/-^ lymphoblasts were incubated with 2-DG over the last 24 h of expansion, washed extensively to remove the inhibitor, and then co-cultured with irradiated splenocytes previously loaded with the OVA_323-339_ peptide. IL-2 **(C)** and cell proliferation **(D)** were determined after 24 h of re-stimulation- Data are means ± SEM (*n*=3). **p<0.01, *p<0.05 (for comparisons between Vhcl- and 2-DG-treated CCR5^-/-^ cells), ^‡^p < 0.05 (for comparisons between Vhcl- and 2-DG-treated WT cells); two-tailed Student *t* test with Holm-Sidak correction for multiple comparisons. **(E)** Model proposed for the regulation of TCR nanoclustering by CCR5. It is here proposed that autocrine/paracrine activation of CCR5 fosters TCR nanoclustering by triggering two independent signals in CD4^+^ T_M_ cells: (i) the inhibition of GATA-1 translocation into the nucleus, and (ii) the stabilization of the glycolytic repressor Bcl-6. More details are available in the text.

Finally, experiments were performed to see whether the metabolic changes driven by CCR5 affect the nanoscopic organization of the TCR in CD4^+^ T cells, a process in which CCR5 signaling is also determinant (6). Electron microscopy (EM) was used to analyze surface replicas of antigen-experienced OT-II WT and CCR5^-/-^ CD4^+^ lymphoblasts after labeling with anti-CD3ϵ antibody and 10 nm gold-conjugated protein A. In agreement with earlier findings (6), TCR nanoclustering was reduced in CCR5^-/-^ compared to WT lymphoblasts (**Figure 5A**). The percentage of monovalent TCRs was significantly higher among CCR5^-/-^ than WT lymphoblasts, whereas the percentage of TCR nanoclusters larger than four TCR molecules was higher in WT than in CCR5^-/-^ cells. To determine if the TCR nanoclustering differences could be ascribed to the enhanced glycolytic activity of CCR5^-/-^ lymphoblasts, TCR organization was analyzed in CCR5^-/-^ lymphoblasts cultured for one day in the presence of the glycolytic inhibitor 2-deoxyglucose (2-DG). 2-DG significantly increased TCR nanocluster number and size in CCR5^-/-^ lymphoblasts, whereas the percentage of monovalent TCRs was drastically reduced (**Figure 5B**). These results suggest that the inhibition of the glycolytic pathway is important in the formation of TCR nanoclusters in antigen-experienced T cells.

The valency of TCR nanoclusters has been related to their sensitivity to antigenic stimulation (5, 6). Given that glycolysis inhibition with 2-DG increased the valency of TCR nanoclusters in CCR5^-/-^ lymphoblasts, tests were made to determine whether 2-DG would increase their antigenic re-stimulation. OT-II WT and CCR5^-/-^ lymphoblasts were incubated for 24 h with 2-DG, and the inhibitor then removed before co-incubation with irradiated splenocytes pre-loaded with different doses of the OVA_323-339_ peptide. Since glycolysis activation is required for complete T cell activation (14–17), IL-2 production and T cell proliferation were analyzed shortly after re-stimulation. IL-2 production was seen to increase significantly in the 2-DG-treated CCR5^-/-^ lymphoblasts compared to controls in an antigen dose-dependent manner (**Figure 5C**). A similar trend (i.e., not significant) towards enhanced IL-2 production was observed in the 2-DG-treated WT lymphoblasts. In contrast, 2-DG treatment tended to inhibit WT and CCR5^-/-^ lymphoblast proliferation at all antigen doses (**Figure 5D**), suggesting that 2-DG interferes with cell proliferation in an antigen- and CCR5-independent manner.

## Discussion

Long-term memory mediated by CD4^+^ T cells is central to the recall response of the adaptive immune system to antigens. CD4^+^ T_M_ cells expand after antigen exposure and begin to make cytokines (which direct immune cell function), provide help in the B cell and CD8^+^ T cell responses, and directly exert effector functions (39, 40). A major characteristic of CD4^+^ T_M_ cells is their ability to respond to lower doses of antigen and/or to reduced levels of co-stimulation compared to naive CD4^+^ T cells (41, 42). This enhanced antigenic sensitivity has been associated with the organization of the TCR into nanoclusters (5, 6) and to the onset of a bioenergetic program in which OXPHOS dominates over glycolysis (43). This work shows CCR5 to be a key regulator of both TCR nanoclustering and the T_M_ cell metabolic program, revealing its importance in the maximization of CD4^+^ T_M_ immune responses (6).

CCR5 deficiency in mice and humans (homozygous carriers for the *ccr5Δ32* polymorphism) causes no significant change in the frequency of the different CD4^+^ T_M_ cell subtypes either in the periphery or in the secondary lymphoid organs. However, the functionality of CCR5-deficient T_M_ cells is lessened, as demonstrated by the reduced expression of cytokines after re-stimulation, or their impaired helper function in the B cell-mediated humoral response (6). This functional deficit is linked to an enrichment of long-chain ceramides in the membrane of antigen-experienced CCR5^-/-^ cells, caused by the increased GATA-1-induced expression of specific ceramide synthases. High levels of ceramides most likely rigidify the plasma membrane, which interferes with TCR nanoclustering and, hence, the ability to respond to low doses of antigen and/or co-stimulation. Here, we show that CCR5^-/-^ T_M_ cells also experience an aberrant increase in glycolysis compared to CCR5-proficient cells. The OXPHOS/glycolytic ratio in the former is reduced, altering the bioenergetic program associated with T_M_ cell differentiation. Importantly, the inhibition of glycolysis increased TCR nanoclustering in antigen-experienced CCR5^-/-^ cells, suggesting a link between high glycolytic activity and reduced TCR nanoclustering. We therefore propose that autocrine/paracrine stimulation of CCR5 enhances TCR nanoclustering in CD4^+^ T_M_ cells through two signals (**Figure 5E**): (i) the reduction of GATA-1 translocation to the nucleus, which restrains the expression of enzymes involved in ceramide biosynthesis (a negative signal for TCR nanoclustering), and (ii) the stabilization of the repressor Bcl-6, which dampens the expression of rate-limiting enzymes for glycolysis (here detected as a negative signal for TCR nanoclustering).

The inhibition of glycolysis seems to be important for the generation of long-lived CD8^+^ T_M_ cells (19, 20), but it also has a negative impact on CD4^+^ and CD8^+^ T cell activation (14–17). This renders it counterintuitive to associate the increased TCR nanoclustering induced by 2-DG treatment in CCR5^-/-^ lymphoblasts with increased sensitivity upon antigen recall. We nonetheless addressed this experimentally by limiting the 2-DG treatment time (to enhance TCR nanoclustering prior to antigen recall), and by analyzing lymphoblast re-stimulation shortly after antigen exposure. This strategy revealed the enhanced production of IL-2 in 2-DG-treated compared to control (vehicle-treated) re-stimulated CCR5^-/-^ lymphoblasts. This occurred in an antigen-dose-dependent manner, suggesting increased antigenic sensitivity in the 2-DG-treated lymphoblasts. The 2-DG treatment also increased IL-2 production in re-stimulated WT lymphoblasts, although the effect was less notable, probably because CCR5 signals already attenuate glycolysis in these cells. In contrast to IL-2, 2-DG treatment impaired WT and CCR5^-/-^ lymphoblast proliferation, another effect of antigenic re-stimulation. The discrepancy between IL-2 and proliferation results might indicate that glycolysis is essential for the generation of the biomass necessary for cell division, but not for the initial TCR-induced signaling involved in IL-2 transcription - at least at the time point analyzed. More studies are needed to confirm this.

The present results suggest that the functional link between CCR5 and Bcl-6 is central for the inhibition of glycolysis in CD4^+^ T_M_ cells. Bcl-6 expression is repressed when IL-2 signaling is elevated (21). Since IL-2 signaling is limited in the T_M_ cell differentiation conditions (driven by IL-15), it is coherent that Bcl-6 levels should be higher in T_M_ than in T_E_ cells, but curiously, this only occurred in CCR5-proficient T_M_ cells. Previous studies have reported no difference in IL-2 expression between CCR5^-/-^ and WT T_M_ lymphoblasts; indeed, IL-2 expression is reduced in antigen-experienced CCR5^-/-^ cells after re-stimulation ( (6) and this study). It is therefore unlikely that the low Bcl-6 levels in CCR5^-/-^ T_M_ lymphoblasts should be a consequence of negative signals provided by autocrine IL-2 production.

How CCR5 signaling induces and/or stabilizes Bcl-6 levels deserves investigation. STAT3 deletion in CD8^+^ T cells reduces Bcl-6 expression during the transition of effector to memory cells (44), and low Bcl-6 levels and impaired T_M_ cell function have been found in humans with dominant-negative STAT3 mutations (45). Since CCR5 triggers STAT signaling (28), CCR5-proficient T_M_ cells might increase Bcl-6 mRNA levels through a STAT3 pathway not operative in CCR5-deficient cells. Nevertheless, preliminary experiments have shown no major differences in phospho-STAT3 levels between WT and CCR5^-/-^ T_M_ cells (data not shown).

An intriguing point is that the enhanced glycolysis of CCR5^-/-^ T_M_ cells is not associated with any increased glucose uptake. Whereas the T_E_ and T_M_ lymphoblasts showed clear divergence in their 2-DG uptake and the expression of some glucose transporters, these differences were independent of CCR5 expression. The upregulation of HK2 and PKM2 might explain the increased glycolytic flow in CCR5^-/-^ T_M_ cells while having no effect on glucose transporter expression. Glucose uptake in lymphocytes might occur by facilitated diffusion through GLUT transporters following a concentration gradient (46). HK2 catalyzes the rate-limiting phosphorylation of glucose to glucose-6-phosphate, which not only provides the initial substrate for glycolysis but removes glucose from equilibrium, favoring its continued diffusion through GLUTs. Pyruvate kinases catalyze the irreversible transphosphorylation between phosphoenolpyruvate and adenosine diphosphate to form pyruvate, another rate-limiting step in glycolysis. There are several isoforms expressed in mammals, which differ in terms of substrate affinity and catalytic efficiency (47). PKM2 is upregulated in cells with a high anabolic profile since its dimeric form is less active. This not only leads to the accumulation of glycolytic intermediates and their diversion to other biosynthetic pathways, but also to the reduced import of pyruvate into the mitochondria.

In summary, CCR5 signaling appears to be a key regulator of glycolytic metabolism specifically in CD4^+^ T_M_ cells. Mechanistically this regulatory activity relies on the stabilization of the glycolytic repressor Bcl-6, which controls the expression of rate-limiting glycolytic enzymes. More important is the observation that the inhibition of glycolysis enhances the valency and frequency of TCR nanoclusters, a factor determining the re-stimulation response of CD4^+^ T_M_ cells to low doses of antigen (6). By hampering the glycolytic pathway and modulating ceramide biosynthesis, CCR5 may foster TCR nanoclustering in CD4^+^ T_M_ cells, making them more efficient in responding to antigen re-exposure. In humans, *ccr5Δ32* homozygosity, which leads to functional CCR5 deficiency, does not cause immunodeficiency but is linked to a greater probability of certain pathogens, including influenza (48), causing fatal infections. Interestingly, CD4^+^ T_M_ cell function seems to be very important in the protective response against these pathogens (40, 49). Thus, CCR5 might decisively maximize CD4^+^ T_M_ cell responses by inhibiting glycolytic metabolism, and increase antigenic sensitivity through the induction of TCR nanoclustering.

## Data Availability Statement

The raw data supporting the conclusions of this article will be made available by the authors, without undue reservation.

## Ethics Statement

The animal study was reviewed and approved by CNB and the Comunidad de Madrid (PROEX 277/14; PROEX 090/19).

## Author Contributions

SM and RB conceived the study.RB, MGdC and RAL designed the metabolic experiments. AM-L performed EM analyses. RB and LR-G performed most of the experiments and interpreted the data. AG-M contributed materials and technical support. ARdM contributed ideas and technical support. SM and RB wrote the manuscript. All authors read, discussed and edited the manuscript. All authors contributed to the article and approved the submitted version.

## Funding

This research was funded by the Spanish Ministerio de Economía y Competitividad (SAF2017–83732-R to SM), the projects PID2020-116303RB-I00/MCIN/AEI/10.13039/501100011033 (to SM) and PID2019-110183RB-C21/MCIN/AEI/10.13039/501100011033 (to AR), the Comunidad de Madrid (B2017/BMD-3733/IMMUNOTHERCAN-CM to SM; P2018/BAA-4343-ALIBIRD2020-CM to AR), and the Merck-Salud Foundation (to SM). AM-L was a recipient of the *Formación del Personal Universitario* predoctoral fellowship from the Spanish Ministry of Education (FPU13/04430).

## Conflict of Interest

The authors declare that the research was conducted in the absence of any commercial or financial relationships that could be construed as a potential conflict of interest.

## Publisher’s Note

All claims expressed in this article are solely those of the authors and do not necessarily represent those of their affiliated organizations, or those of the publisher, the editors and the reviewers. Any product that may be evaluated in this article, or claim that may be made by its manufacturer, is not guaranteed or endorsed by the publisher.
