# Supplementary material for: The Chemokine Receptor CCR5 Links Memory CD4+ T Cell Metabolism to T Cell Antigen Receptor Nanoclustering
Source: Front Immunol. 2021 Dec 7;12:722320. doi: 10.3389/fimmu.2021.722320 (PMC8688711; doi:10.3389/fimmu.2021.722320)
Supplement: Supplementary file 1 [file DataSheet_1.pdf]

The chemokine receptor CCR5 links memory CD4$^+$ T cell metabolism to T cell antigen receptor nanoclustering

Raquel Blanco[1], Marta Gómez de Cedrón, Laura Gámez-Reche, Ana Martín-Leal, Rosa A. Lacalle, Alicia González-Martín, Ana Ramírez de Molina, Santos Mañes

.

Supplementary Material

Supplementary Figures S1-S7

Supplementary Table S1

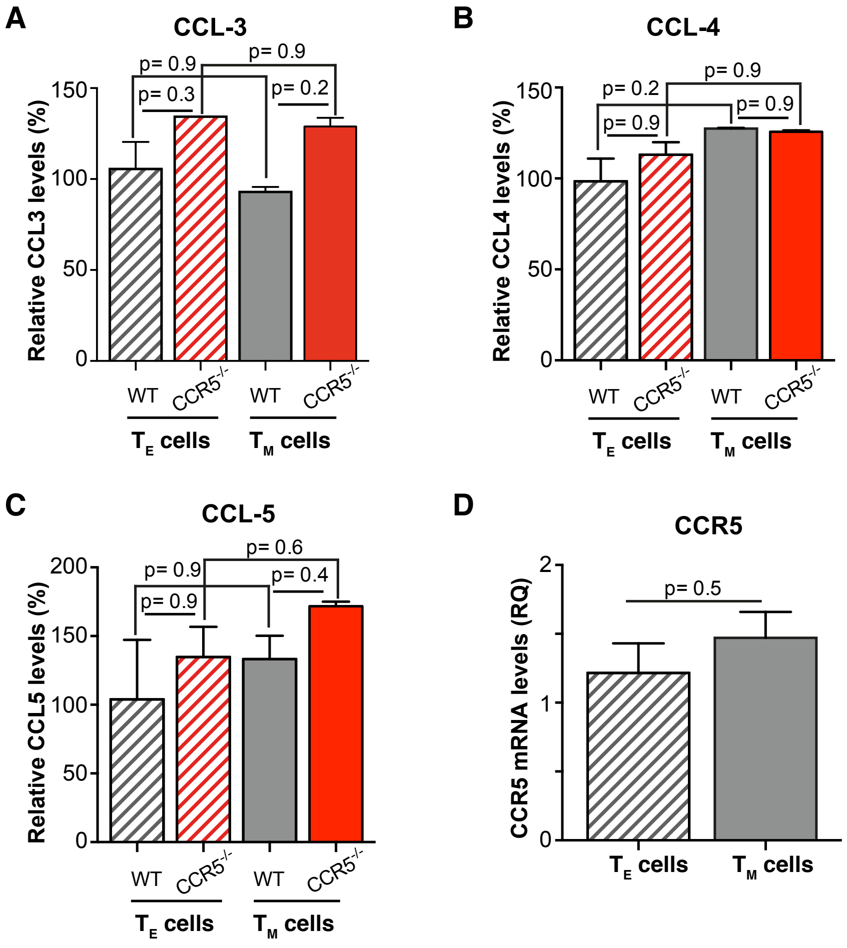

Supplementary Figure S1. CCR5 ligands and receptor levels in CD4^{+} T_E and T_M cells. A-C. CCL3, CCL4 and CCL5 levels in the supernatant of IL-2- (T_E; hatched bars) or IL-15-expanded (T_M; solid bars) WT and CCR5$^{-/-}$ lymphoblasts, as determined by ELISA. Values are expressed as relative levels using IL-2-expanded WT cells as a reference (n = 3). **D**. Relative CCR5 mRNA expression in T_E and T_M WT lymphoblasts. P-values for the indicated comparisons are shown; two-way ANOVA with Bonferroni post hoc test (*A-C*) or the unpaired two-tailed Student's *t* test with Welch's correction (*D*).

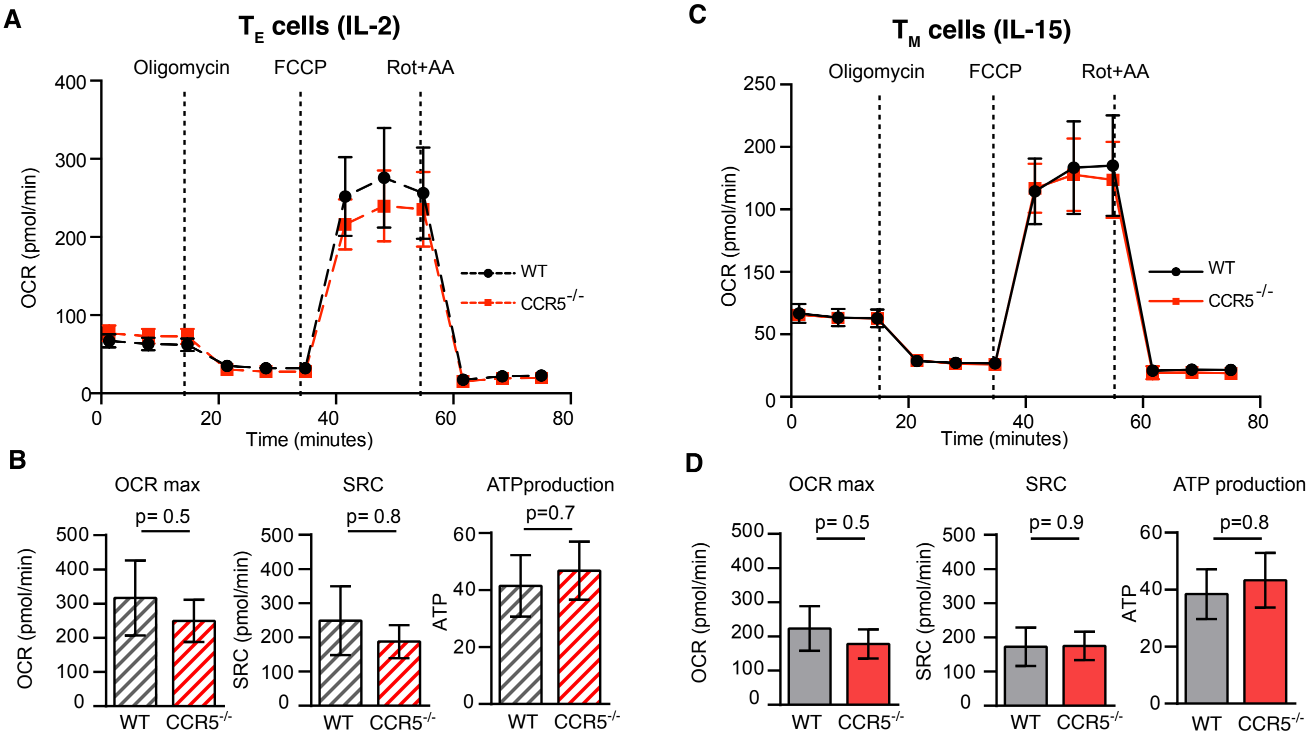

Supplementary Figure S2. CCR5 does not affect mitochondrial metabolism in CD4$^+$ T_E or T_M cells. A-D. Oxygen consumption rate (OCR) profiles of IL-2- (*A*, *B*) or IL-15-expanded WT and CCR5$^{-/-}$ lymphoblasts (*C*, *D*) using the mitochondrial stress test and glucose as a carbon source. OCR was recorded under basal conditions and after sequential addition of the ATP synthase (complex V) inhibitor oligomycin, the mitochondrial uncoupler FCCP, and the complex I and III inhibitors antimycin A/rotenone (Rot+AA). OCR profiles were used to calculate (*B*, *C*): maximum OCR (OCR max) obtained after FCCP addition, spare respiratory capacity (SRC) calculated as the difference between maximum and basal OCR, and mitochondrial-linked ATP production calculated upon the addition of oligomycin. Data are means±SEM ($n \geq 9$ from three independent experiments). P-values for the indicated comparisons are shown (two-tailed Student's *t* test).

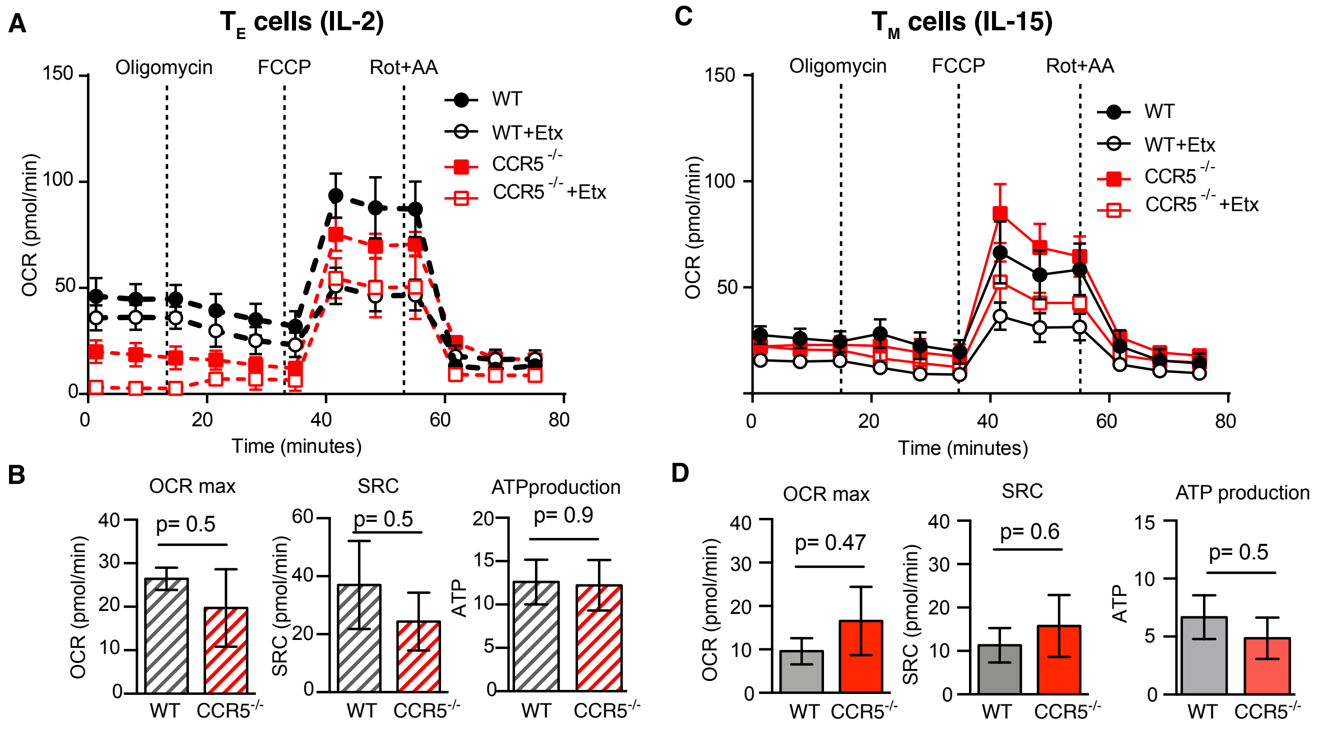

Supplementary Figure S3. CCR5 does not affect FAO in either CD4$^+$ T$_E$ or T$_M$ cells. A-D. IL-2-(T$_E$; *A, B*) and IL-15-expanded (T$_M$; *C, D*) WT and CCR5$^{-/-}$ OT-II lymphoblasts were treated with etomoxir or vehicle and OCR-analyzed using palmitate as the carbon source. OCR was recorded under basal conditions and after the sequential addition of oligomycin, FCCP, and antimycin A/rotenone (Rot+AA). The OCR values obtained for vehicle-treated cells (solid symbols) were higher than those obtained with etomoxir-treated cells (open symbols), allowing FAO-specific OCR values to be calculated (*A, C*). FAO-specific OCR values were used to calculate maximum OCR, SCR and mitochondrial-linked ATP production in the different cell types analyzed (*B, D*). Data are mean ± SEM. The p-values for the indicated comparisons are shown (two-tailed Student's *t* test).

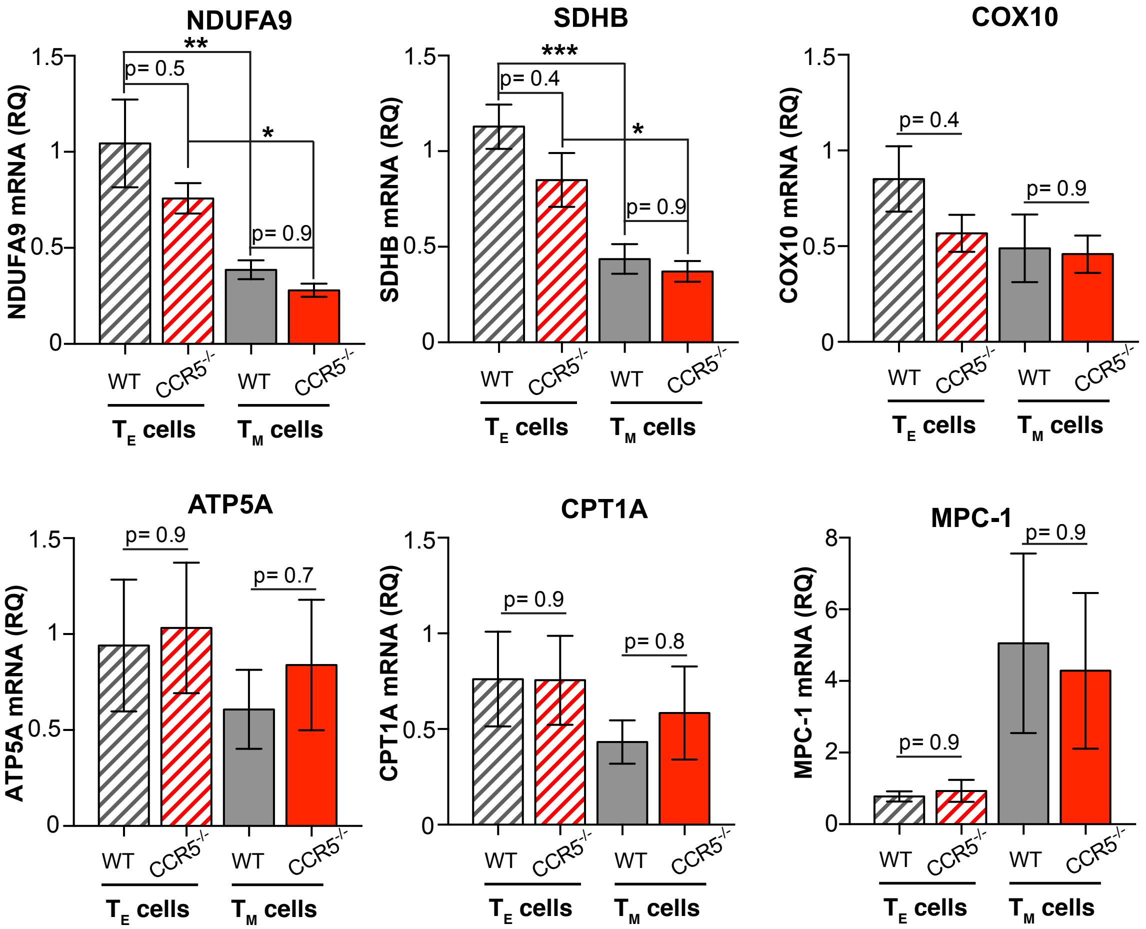

Supplementary Figure S4. CCR5 expression does not affect mitochondrial/OXPHOS metabolic genes. Relative mRNA expression of NDUFA9 (respiratory complex I), SDHB (respiratory complex II), COX10 (respiratory complex IV), ATP5A1 (respiratory complex V), CPT1A (fatty acid oxidation), MPC-1 (pyruvate uptake into mitochondria) in WT (gray) and CCR5$^{-/-}$ (red) TM and TE lymphoblasts (n = 6). Data are means±SEM. ***p<0.001, **p<0.01, *p<0.05, two-way ANOVA with Bonferroni post-hoc test.

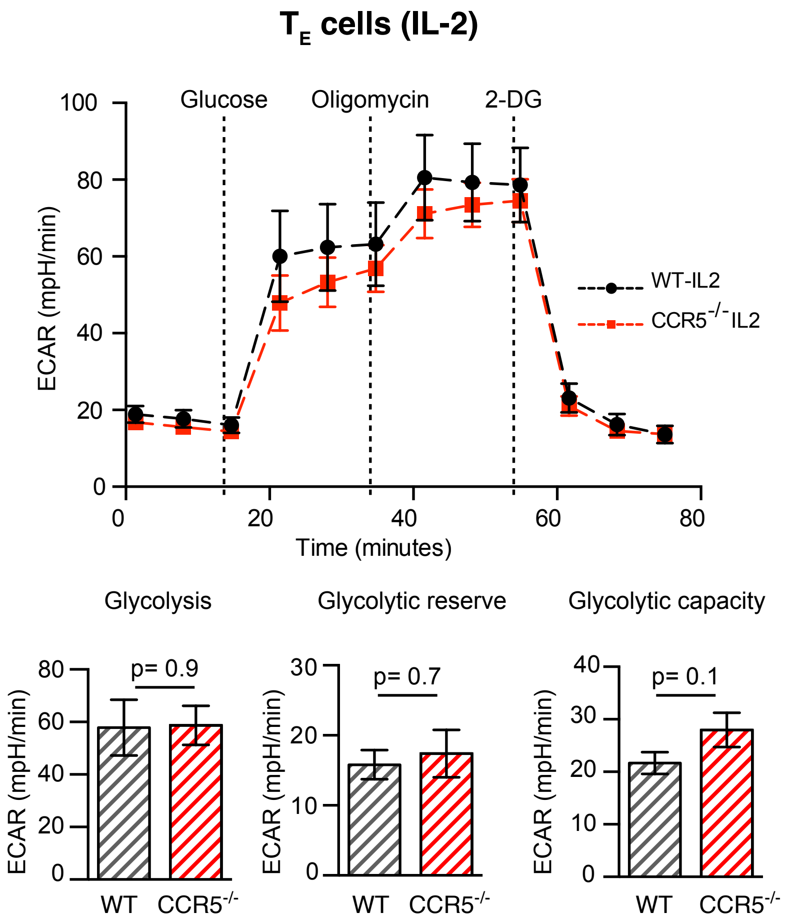

Supplementary Figure S5. CCR5 does not affect glycolytic metabolism in CD4$^+$ T$_E$ cells. WT and CCR5$^{-/-}$ T$_E$ (IL-2-expanded) lymphoblasts were incubated in XF assay medium supplemented with 2 mM glutamine and 1 mM sodium pyruvate, with subsequent additions of glucose, oligomycin and 2-DG, as indicated. Top: ECAR profiles for WT and CCR5$^{-/-}$ T$_E$ lymphoblasts. Bottom: determination of glycolysis, glycolytic reserve and glycolytic capacity from the ECAR profile data (see text for details). Data are means±SEM ($n \geq 9$ from three independent experiments). P-values for the indicated comparisons are shown (two-tailed Student's t test).

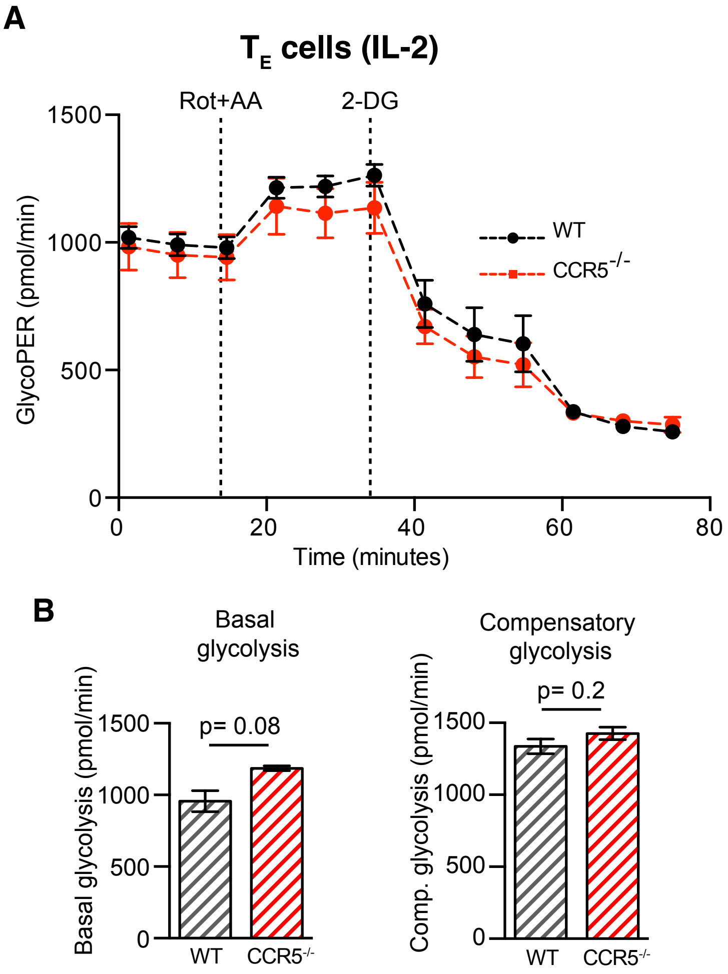

Supplementary Figure S6. CCR5 does not affect the glycolytic proton efflux rate in CD4^{+} T$_{E}$ cells. A. GlycoPER profiles in IL-2-expanded WT and CCR5$^{-/-}$ lymphoblasts under basal conditions (non-buffered XF assay medium pH 7.4, containing 25 mM glucose and 2 mM glutamine and 1 mM sodium pyruvate, in the absence of CO_2), and following the addition of rotenone/antimycin A (Rot+AA) and 2-DG. **B.** Determination of basal and compensatory glycolysis (*C*) from GlycoPER values as in *A*. Data are mean ± SEM. The p-values for the indicated comparisons are shown (two-tailed Student's *t* test).

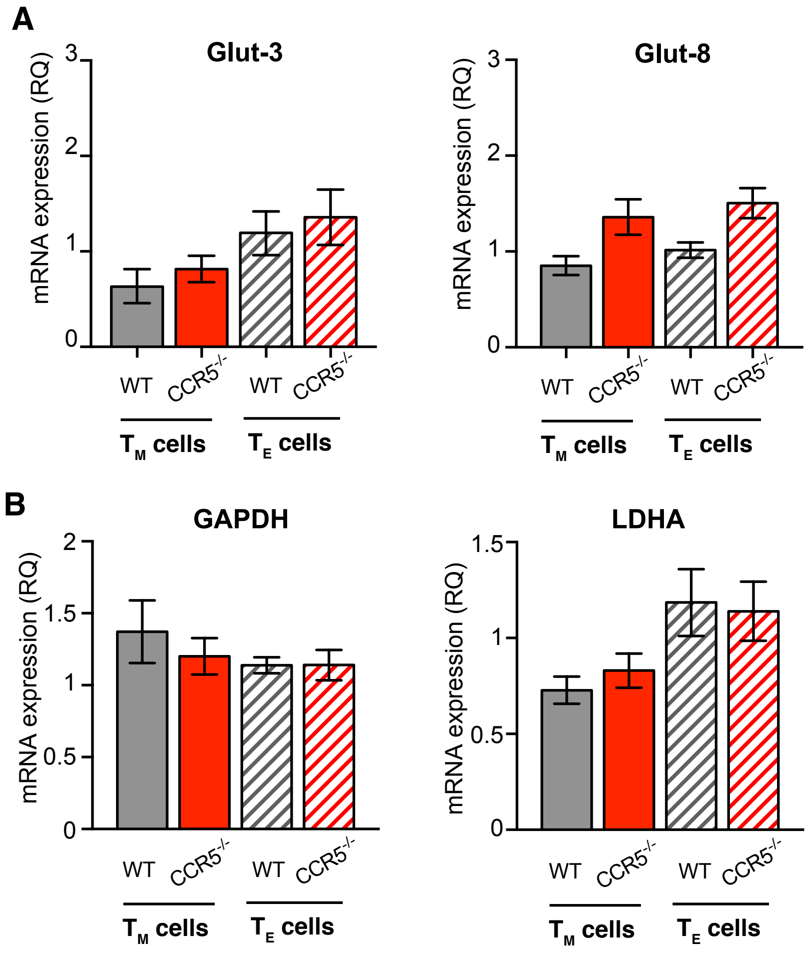

Supplementary Figure S7. Glucose transporters and glycolytic enzymes not affected by CCR5 expression or CD4^{+} T cell differentiation state. A, B. Relative mRNA expression of the glucose transporters Glut-3 and Glut-8 (*A*), and the glycolytic enzymes GAPDH and LDHA (*B*), in WT (gray) and CCR5$^{-/-}$ (red) T_M (solid) and T_E (hatched) lymphoblasts (n = 12). Data are mean ± SEM. Differences did not reach statistical significance (two-way ANOVA with Bonferroni post-hoc test).

Supplementary Table S1. List of primers used for RT-qPCR analyses

Gene Symbol	Forward primer (5’->3’)	Reverse primer (5’->3’)
GLUT1	ACGATCTGAGCTACGGGGT	CCTCCCACAGCCAACATGAG
GLUT3	TAAACCAGCTGGGCATCGTTGTTG	AATGATGGTTAAGCCAAGGAGCCC
GLUT6	TTGGTGCTGTGAGGCT	TGGCACAAACTGGACGTA
GLUT8	TTCATGGCCTTTCTAGTGACC	GAGTCCTGCCTTTAGTCTCAG
HK2	CTGAGCAAGGAGACGCATGA	GCAGGACCCGGAAGTTTGTT
GAPDH	AGGTCGGTGTGAACGGATTTG	TGTAGACCATGTAGTTGAGGTCA
LDHA	GCACTGACGCAGACAAGG	TGATCACCTCGTAGGCACTG
PKM2	GCAGTGGGGCCATTATCGT	TCAGCACGGCATCCTTACAC
BCL-6	CCGGCTCAATAATCTCGTGAA	GGTGCATGTAGAGTGGTGAGTGA
CCR5	TCCGTTCCCCCTACAAGAGA	TTGGCAGGGTGCTGACATAC
NDUFA9	AGGCATTGTGGGAGAAGATG	CCTGTAGCCCCAAACACAGT
SDHB	AATTTGCATTTACCGATGGGA	AGCATCCAACACCATAGGTCC
COX10	AGAAGAQGCTATACAGGGATTGCC	CTGTGTGACATACATGCGCTT
ATP5A1	CACAGCTGAGATGTCCTCCA	CATTGTCGGGTTCCAAGTTC
CPT1A	CTCCGCCTGAGCCATGAAG	CACCAGTGATGATGCCATTCT
MPC1	TTCGCCCTCTGTTGCTATTC	GAGCTGAGCTACTTCGTTTGT
18S rRNA	GAGAAACGGCTACCACATCC	GGGTCGGGAGTGGGTAAT
